# Analysis of the “centrosome-ome” identifies MCPH1 deletion as a cause of centrosome amplification in human cancer

**DOI:** 10.1038/s41598-020-68629-4

**Published:** 2020-07-17

**Authors:** Ryan A. Denu, Mark E. Burkard

**Affiliations:** 1 Division of Hematology/Oncology, Department of Medicine, School of Medicine and Public Health, University of Wisconsin-Madison, 6059 WIMR, 1111 Highland Avenue, Madison, WI 53705 USA; 2 Carbone Cancer Center, University of Wisconsin, Madison, WI USA; 3 Medical Scientist Training Program, School of Medicine and Public Health, University of Wisconsin-Madison, Madison, WI USA

**Keywords:** Cancer genomics, Cell signalling, Mechanisms of disease

## Abstract

The centrosome is the microtubule organizing center of human cells and facilitates a myriad of cellular functions including organization of the mitotic spindle to ensure faithful chromosome segregation during mitosis, cell polarization and migration, and primary cilia formation. A numerical increase in centrosomes, or centrosome amplification (CA), is common in cancer and correlates with more aggressive clinical features and worse patient outcomes. However, the causes of CA in human cancer are unclear. Many previous studies have identified mechanisms of CA *in cellulo*, such as overexpression of PLK4, but it is unclear how often these are the primary mechanism in human disease. To identify a primary cause of CA, we analyzed The Cancer Genome Atlas (TCGA) genomic and transcriptomic data for genes encoding the 367 proteins that localize to the centrosome (the “centrosome-ome”). We identified the following candidates for primary causes of CA: gain-of-function alterations of CEP19, CEP72, CTNNB1, PTK2, NDRG1, SPATC1, TBCCD1; and loss-of-function alterations of CEP76, MCPH1, NEURL4, and NPM1. *In cellulo* analysis of these candidates revealed that loss of MCPH1/microcephalin caused the most robust increase in centriole number. MCPH1 deep gene deletions are seen in 5–15% of human cancers, depending on the anatomic site of the tumor. Mechanistic experiments demonstrated that loss of MCPH1 caused a CDK2-dependent increase in STIL levels at the centrosome to drive CA. We conclude that loss of MCPH1 is common in human cancer and is likely to be a cause of CA.

## Introduction

The centrosome is the microtubule-organizing center of human cells. A numerical increase in centrosomes, or centrosome amplification (CA), is common in cancer and correlates with more aggressive clinical features and worse patient outcomes^1,2^. Previous studies have established *in cellulo* models of CA by overexpression of PLK4^3^ or modulating other centrosome genes^4^. Although this identifies many genes as candidates for underlying causes of CA, the actual clinically-relevant molecular mechanisms driving CA in human cancer have not been determined.

CA is thought to arise by two major mechanisms: (1) centriole overduplication and (2) cell doubling events, each of which could be further subdivided into those with and without a primary genetic cause. We previously determined that the former, centriole overduplication, is primarily responsible for CA in human melanoma^5^. Additional evidence comes from centriole analysis of human cancer cell lines, demonstrating that only a subpopulation of cell lines with CA have an increase in ploidy, suggesting different origins of ploidy and CA^6^. We therefore sought to identify potential primary genetic mechanisms leading to centriole overduplication in human cancer. To address this question, we analyzed genomic and transcriptomic alterations in 367 centrosome proteins using TCGA data from 10,207 independent cancers representing 22 of the most common tumor sites. We identified a list of candidate centrosome genes that are most frequently altered in cancer and tested them *in cellulo* to determine the predominant causes of centriole overduplication in human cancer.

## Methods

### Bioinformatic analysis of centrosome genes

A list of all 367 centrosome genes (Supplemental Table 1) was compiled by searching for “centrosome” on Uniprot and supplementing with proteins discovered at the centrosome in previous proteomic analysis of isolated centrosomes^7^. Data were extracted from the following 22 TCGA datasets using cBioPortal^8^, selected based on their size and availability of associated clinical data: acute myeloid leukemia, 200 patients; breast carcinoma, 1,105; melanoma, 478; colorectal carcinoma, 633; glioblastoma, 607; low grade glioma, 532; ovarian serous adenocarcinoma, 607; lung adenocarcinoma, 522; lung squamous cell carcinoma, 504; prostate adenocarcinoma, 499; head and neck squamous cell carcinoma, 530; renal clear cell carcinoma, 538; stomach adenocarcinoma, 478; bladder urothelial carcinoma, 413 patients; hepatocellular carcinoma, 442; cervical carcinoma, 309; endometrial carcinoma, 373; esophageal adenocarcinoma, 186; pancreatic adenocarcinoma, 186; pheochromocytoma and paraganglioma, 184; sarcoma, 365; and thyroid carcinoma, 516. This yielded a total of 10,207 cancers. We analyzed the percent of cancers with mutations, copy number variations, and altered gene expression (mRNA Z score < 2 or > 2) for each of the 367 centrosome genes. Each data set (e.g. breast, prostate, etc.) was weighted equally during analyses in order to be able to detect certain tissue-specific alterations that may give rise to CA. Data were not available for the following centrosome genes: BOD1L2, PTPN20, ROSSF10, STARD9, TSSK2, WASH1.

To assess centrosome gene alterations more common in TP53 mutated/deleted cancers, we excluded the tumors associated with viruses that have been shown to inhibit p53 function: (head and neck, liver, and cervical). We then calculated the fold enrichment of alterations in each centrosome gene in p53 mutated/deleted cancers versus p53 wild-type cancers. Genes were excluded if their alterations were not enriched in p53 mutated/deleted cancers. We also supplemented our list of hits with the following three centrosome genes identified by MutSigCV^9^, which identifies genes that are mutated more often than expected by chance: CEP76, CTNNB1, and NPM1.

### Cell culture

CAL-51, DLD-1, HCT116, HeLa, MCF10A, MDA-MB-231, MDA-MB-453, MDA-MB-468, PC3, Phoenix, RPE, and 293T cell lines were obtained from ATCC. All cell lines were authenticated by STR analysis using the Promega PowerPlex 16 HS System Kit (DC2101) at the University of Wisconsin Translational Research Initiatives in Pathology (TRIP) laboratory. Cells were propagated at 37 °C in 5% CO_2_ and grown in basal medium supplemented with 10% fetal bovine serum and 100 units/mL penicillin–streptomycin. The basal medium used for each cell line is as follows: high glucose DMEM for HeLa, PC3, Phoenix, and 293T; RPMI-1640 for DLD-1; and McCoy’s 5a for HCT116. MCF10A cells were grown in 1:1 DMEM and Ham’s F-12 supplemented with horse serum, EGF, hydrocortisone, cholera toxin, and 100 units/mL penicillin–streptomycin.

Lentivirus encoding shRNAs were produced by transfecting a T25 of 293T cells with 2 µg pLKO.1 vector, 1 µg pVSV-G, and 1 µg psPAX2, and Fugene (Promega). For stable retroviral transduction, a T25 of Phoenix cells was transfected with 2 µg pSuperior.retro.puro construct and 1 µg pVSV-G. For both lentivirus and retrovirus, fresh medium was applied at 24 h post-transfection, and virus was harvested 24 h later, clarified by centrifugation and filtration through a 0.45 μm membrane to remove cell debris, and diluted 1:1 with complete medium containing 10 μg/mL polybrene. Target cells (HeLa) at 40–60% confluence were transduced for 24 h, then given fresh media for 24 h, then 1 µg/mL puromycin was added for 4 days. Fresh media was added for 24 h before using the cells for subsequent experiments.

To overexpress cDNAs of centrosome candidate genes, HeLa cells were plated in 6-well plates, grown to 80% confluence, and transfected with 2 µg cDNA in pcDNA5 using Fugene (Promega). Cells were given fresh media 12 h after the transfection. 24 h after the transfection, cells were either transferred to coverslips for fixation and immunofluorescence or harvested for qRT-PCR to assess transfection efficiency.

Chemicals used in this study include aphidicolin (5 µM, Sigma), doxycycline (2 µg/mL, Thermo Fisher), puromycin (1 µg/mL for HeLa cells, Thermo Fisher), CHIR-124 (100 nM, MedChemExpress), milciclib (100 nM, MedChemExpress), centrinone B (500 nM, Tocris), thymidine (2 mM, Thermo Fisher), and RO-3306 (10 µM, Tocris).

### Molecular biology

The following cDNAs were acquired: NPM1 was cloned from HeLa cDNA library, MCPH1 was from Shiaw-Yih Lin (Addgene plasmid #16205), STIL was from transOMIC (BC16223), PTK2 was from Michael Davidson (Addgene plasmid #55122), NDRG1 was from Ben Park (Vanderbilt University), CTNNB1 wild-type and S33Y were from Eric Fearon (Addgene plasmids #16828 and #19286), and CEP19 (GE MHS6278-202828648), SPATC1 (GE MHS6278-202802271), and TBCCD1 (GE MHS6278-202801247) were from GE Healthcare. These cDNAs were cloned into pcDNA5/FRT/TO with C-terminal mNeonGreen tag (vector provided by Jun Wan and Beth Weaver, University of Wisconsin). Sequencing was performed to verify cloning fidelity (Protein CT and Quintara Biosciences). All constructs were full-length cDNAs unless otherwise noted.

Mutant NPM1 (W288Cfs*12 insertional frameshift mutation, which is predicted to mutate the remaining C-terminal amino acids of the protein and increase the length of the protein by 10 amino acids) was cloned from a HeLa cDNA library using the following primers: forward 5′CCCCGGATCCATGGAAGATTCGATGGACATGG, reverse: 5′ CCCCGAATTCCTATTTTCTTAAAGAGACTTCCTCCACTGCCAGACAGAGATCTTGAATAGCCTCTTGGT.

To make shRNA vectors, shRNAs targeting sequences were cloned into pLKO.1-TRC, a kind gift from David Root (Addgene plasmid #10878). The following sequences were used for initial screening: CEP76, 5′ ACGACTTTAGTAGCATCATAT; MCPH1, AGGAAGTTGGAAGGATCCA; NEURL4, 5′ CCATCATGCAAGACGGTAATT; NPM1, 5′ TTACGAAGGCAGTCCAATTAA; non-targeting control, 5′ CCGCAGGTATGCACGCGT (kind gift from David Root, Addgene plasmid #10879). Lentivirus was produced as described above. Efficiency of gene depletion was assessed by qRT-PCR. Primer sequences are available in Supplemental Table 4.

To make doxycycline-inducible shRNA cell lines, the MCPH1 targeting sequences were cloned into pSuperior.retro.puro following the manufacturer’s protocol (OligoEngine). Two different shRNAs were used for MCPH1: 5′ AGGAAGTTGGAAGGATCCA and 5′ CTCTCTGTGTGAAGCACCT. Retrovirus was made and used to transduce HeLa cells stably expressing TetR (kind gift from Jun Wan and Beth Weaver, University of Wisconsin), as described above.

To genetically inhibit CDK2, pcDNA3-myc3-p27 (a gift from Yue Xiong, Addgene plasmid #19937) was obtained and transfected into HeLa cells using Fugene (Promega).

### Immunofluorescence (IF) and microscopy

IF and imaging were carried out as previously described^5^. Cells were seeded on glass coverslips in 24-well plates and fixed with 100% ice-cold methanol for 15 min. Fixed cells were then blocked for 30 min in 3% bovine serum albumin (BSA) and 0.1% triton X-100 in PBS (PBSTx + BSA). Primary antibodies were incubated in PBSTx + BSA for 1 h at room temperature and washed three times in PBSTx, followed by secondary antibody incubation in PBSTx + BSA for 30 min at room temperature and two washes with PBSTx. Cells were counterstained with DAPI and mounted on glass slides with Prolong Gold antifade medium (Invitrogen). Image acquisition was performed on a Nikon Eclipse Ti inverted microscope with 60 × and 100 × objectives and Hamamatsu ORCA Flash 4.0 camera. For images displayed in the figures, optical sections were taken at 0.2-μm intervals and deconvolved using Nikon Elements. Where appropriate, the observer was blinded to treatment condition during image acquisition and analysis. Images were processed and analyzed using Nikon Elements.

Primary antibodies used were: centrin (Millipore, 04–1624), γ-tubulin (Abcam, ab27074), pericentrin (Abcam, ab4448), CEP164 (gift from Erich Nigg), β-actin (DSHB, JLA20, deposited by Lin, J.J.-C.), α-tubulin (DSHB, 12G10, deposited by Frankel, J./Nelsen, E.M.), FLAG (Sigma, F1804), human autoantibody against centromere (ImmunoVision, HCT-0100), MCPH1 (kind gift from Hui Dai and Shiaw-Yih Lin), PLK4 (Abcam, ab137398), STIL (Bethyl, A442A), and CDK2 (BD Biosciences, 610145). Alexa fluor-conjugated secondary antibodies were used (Invitrogen, 1:350).

### Quantitative reverse transcriptase polymerase chain reaction (qRT-PCR)

RNA was isolated from cells using TRI Reagent (Molecular Research Center). DNase treatment was used to remove DNA contamination. RNA was then converted to cDNA using the Quantitect Reverse Transcription Kit (Qiagen). A StepOne Plus (Applied Biosystems) real-time PCR thermal cycler was used for amplification. The average C_T_ value of three housekeeping genes (*RRN18S, GAPDH *and* ACTB*) was subtracted from each experimental C_T_, then $2^{{ - \Delta {\text{C}}_{{\text{T}}} }}$ values were normalized to controls (either vector only control or scrambled shRNA control) and compared. Additionally, the ΔΔC_T_ method was employed to calculate the fold change in gene expression. Primer sequences are provided in Supplemental Table 4.

### Immunoblotting

Cells were lysed in lysis buffer (50 mM HEPES, pH 7.5, 100 mM NaCl, 0.5% NP-40, 10% glycerol) containing phosphatase inhibitors (10 mm sodium pyrophosphate, 5 mM β-glycerol phosphate, 50 mm NaF, 0.3 mm Na_3_VO_4_), 1 mM PMSF, protease inhibitor cocktail (Thermo Scientific), and 1 mm dithiothreitol. Samples were sonicated and heated in SDS buffer. Proteins were separated by SDS-PAGE, transferred to PVDF membrane (Millipore), and blocked for at least 30 min in 5% milk and 0.1% Tween 20 in Tris-buffered saline, pH 7.4 (TBST + milk). Membranes were incubated 1 h at room temperature or overnight at 4 °C with primary antibodies diluted in TBST + 5% milk, washed three times with TBST, and incubated for 1 h at room temperature in secondary antibodies conjugated to horseradish peroxidase in TBST + 5% milk. Membranes were washed and developed with luminol/peroxide (Millipore) and visualized with film.

Antibodies utilized for immunoblotting include: actin (DSHB, JLA20, 1:1,000), CEP131 (Thermo, PA5-38978, 1:1,000), FLAG (Sigma, F1804, 1:5,000), GFP (Invitrogen, A-11120, 1:1,000), and alpha tubulin (DSHB, 12G10, 1:1,000). HRP-conjugated secondary antibodies were used (Jackson, 1:5,000). Relative intensities of bands were calculated using ImageJ from scanned images and normalized to their respective loading control intensity.

### Kinase assays

Cell extracts from a nearly confluent T25 flask were lysed in buffer (see above) and incubated with 1 µg of either anti-CDK2 antibody (BD Biosciences, 610145) or anti-GFP antibody (DSHB, 12A6; used as a negative control) overnight. Antibody–antigen complexes were precipitated using protein A and G sepharose beads (GE Healthcare). Bead complexes were washed three times with lysis buffer. Kinase assay reactions were incubated at 30 °C for 30 min in buffer (50 mM Tris–HCl, pH 7.5, 10 mM MgCl_2_, 0.1 mM NaF, 10 μM Na_3_VO_4_) with 1 mM DTT, 1 μM cold ATP, 2 μCi [γ-^32^P] ATP, and 4 μg histone H1 (Sigma, H1917). Reactions were resolved by SDS-PAGE. Dried gels were exposed to a storage phosphor screen (GE Healthcare), and γ-^32^P incorporation was visualized by Typhoon TRIO imager (GE Healthcare).

### Statistics

Statistical evaluations were performed using Prism (Graphpad Software) or Microsoft Excel. Two-tailed *t* tests and one-way ANOVA were used for comparisons. For multiple comparisons, Tukey’s correction was made. Survival analysis was performed using the Kaplan–Meier method. P-values < 0.05 were considered significant for all tests, and designations are made in the figures for statistical significance. For figures displaying data from individual cells, “SuperPlots” were made^10^. A datapoint is shown for each cell, and the average of the cells in an independent experiment is overlaid with larger outlined dot. Each independent experiment is coded with a different color. Bars represent means ± SEM from three independent experiments.

## Results

### Survey of centrosome gene alterations in human cancer

**Figure 1:**
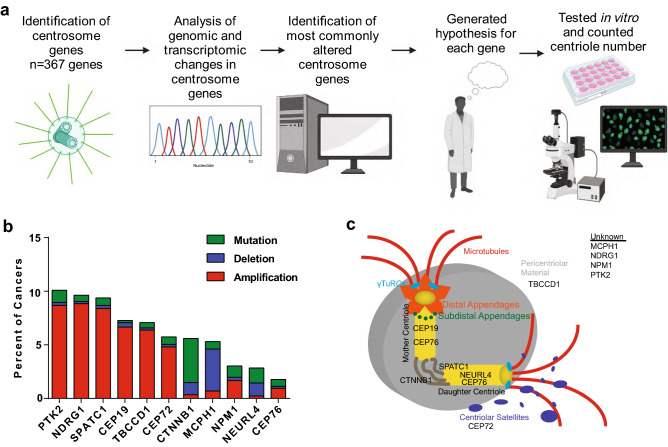
Bioinformatic analysis of the “centrosome-ome”. (**a**) Schema for determining the centrosome genes most commonly altered in human cancer. BioRender was used to make this panel. (**b**) Bar graph showing the percent of cancers with genomic alterations in the most altered centrosome genes from our in silico screen. (**c**) Diagram of the centrosome demonstrating the localization, if known, of the most commonly altered centrosome genes identified by our in silico screen.

The goal of this project was to identify genetic causes of CA in human cancer. We began by testing the hypothesis that CA is due to alterations in centrosome genes. To systematically examine the “centrosome-ome” in human cancer, we interrogated publicly-available genomic and transcriptomic datasets. We analyzed the 367 proteins that localize to the centrosome (listed in Supplemental Table 1) using TCGA data. We assessed mutations, copy number alterations, and gene expression changes and compiled a list of the most commonly altered centrosome genes (Fig. 1a). Given that cells with CA arrest unless other compensatory alterations are made, such as loss of p53^11^ or p21^12,13^, we ranked alterations in centrosome genes by the fold enrichment in p53/p21 mutant versus p53/p21 wild-type tumors (Supplemental Table 2) and excluded genes if their alterations were not enriched (Fig. 1a). Lastly, the PIDDosome is also important for cell cycle arrest in the presence of CA^14^, so we also considered centrosome gene alterations in tumors with mutant PIDDosome proteins; however, mutations and deletions in these PIDDosome genes are not commonly seen in human cancer and were not incorporated to select our list of candidate genes. Lastly, we compared our list to those found on MutSigCV to be significantly mutated in cancer from a previous study, which incorporated high mutational burden relative to background expectation, clustering of mutations within a gene, enrichment of mutations in evolutionarily conserved sites^9^. This list of commonly mutated cancer genes contained 3 centrosome genes: CEP76, CTNNB1, and NPM1, which were included in our analysis.

Taking all these data into account, we identified the following candidates as potential causes of CA in human cancer: gain of function of CEP19, CEP72, CTNNB1, PTK2, NDRG1, SPATC1, TBCCD1; and loss of function of CEP76, MCPH1, NEURL4, NPM1. The rationale for each of these selections is described in Table 1. A summary diagram of alterations of these genes is shown in Fig. 1b, their centrosome localization, if known, is shown in Fig. 1c. Interestingly, there is a strong co-occurrence of genomic amplifications of NDRG1, PTK2, and SPATC1; all three of these genes are on chromosome 8q. Gain of chromosome 8q is associated with poorer prognosis in multiple human cancers. This is thought to be due to amplification of the proto-oncogene C-MYC, which is on chromosome 8q^15^.

**Table 1:** Top hits for drivers of centrosome amplification in human cancer.

Centrosome gene	Known functions (especially centrosome-related functions)	Hypothesis
CEP19	Ciliation; microtubule anchoring to the centrosome; inactivation results in morbid obesity	Gain of function causes CA
CEP72	Centriolar satellite component; recruit key centrosomal proteins to centrosome; microtubule nucleation; BRCA1 interactor; overexpression increases CIN, aneuploidy, lagging chromosomes	Gain of function causes CA
CEP76	Limits centriole duplication; depletion drives aberrant amplification of centrioles	Loss of function causes CA
CTNNB1	Wnt signaling pathway; negative regulator of centrosome cohesion; overexpression of stabilized mutant increases centrosomes	Gain of function causes CA
MCPH1	Neurogenesis; chromosome condensation; DNA damage response; restrains DNA damage-induced CA; germline mutations cause microcephaly	Loss of function causes CA
NEURL4	Interacts with CP110 (important for limiting centriole elongation)	Loss of function causes CA
NDRG1	Cell trafficking; regulates centrosome number	Gain of function causes CA
NPM1	Ribosome biogenesis; depleting NPM1 results in CA	Loss of function causes CA
PTK2	Cell migration, adhesion, spreading, actin reorganization, focal adhesion formation, proliferation, apoptosis	Gain of function causes CA
SPATC1	Proximal centriole marker	Gain of function causes CA
TBCCD1	Localizes to a region sub-proximal to centrioles; mutant cells have variable numbers of centrioles and centriole positioning defects required for mother-daughter centriole linkage and mitotic spindle orientation; migration	Gain of function causes CA

In general, point mutations in centrosome genes were rare. The most common single point mutation was found in NPM1 (W288Cfs*12 insertional frameshift mutation), which is found exclusively in acute myeloid leukemia (AML), and causes a frameshift mutation at the C-terminus. Patients with this mutation have worse outcomes (Supplemental Fig. 1). The effects of this mutation on centrosome/centriole number have not been reported. To evaluate NPM1, we generated the mutant cDNA that would be produced with this frameshift mutation and overexpressed it in HeLa cells. However, there was no significant difference in centriole number between cells with mutant and wild-type NPM1 (Supplemental Fig. 1). Further, we find a lack of coincidence of NPM1 mutations with compensatory changes in p53, p21 and PIDDosome genes (Supplemental Fig. 1); further, there is a significant mutual exclusivity of NPM1 mutations and p53 alterations (Bonferroni corrected p value < 0.001). We conclude that NPM1 mutations are not likely to drive CA when they are found in AML.

We initially focused on centrosome genes that are required for centriole duplication: PLK4, STIL, CEP152, CEP192, TUBE1, CETN2, CPAP, SAS6, CEP135 (Supplemental Table 3). CEP152 and CEP192 recruit PLK4, then PLK4 binds STIL, which activates PLK4 activity. PLK4 then phosphorylates STIL, allowing STIL to bind SAS6. CEP120, CPAP/CENP-J, SPICE-1 are then recruited, followed by CEP135, POC1A, POC1B, POC5, CNTROB, and CP110, which allow for elongation. Interestingly, there was a relative dearth of alterations in these genes, perhaps suggesting that centriole duplication is crucial to the vitality of cancer cells. Recent evidence has demonstrated that some cancer cell lines have over-elongated centrioles, which can promote CA^6^. Therefore, we hypothesized that CP110 loss-of-function may drive CA; however, mutations or genomic deletions were not commonly seen in CP110 (Supplemental Fig. 2). Similarly, CPAP/CENP-J/SAS4 is important for centriole elongation, so we hypothesized that CPAP gain-of-function may drive CA; however, gain-of-function mutations or genomic amplifications were rarely seen (Supplemental Fig. 2). STIL was recently reported to be a substrate of SCF-βTrCP-mediated degradation, and mutations in the DSG domain (residues 394–399) prevent such degradation, leading to CA^16^. However, mutations in this DSG motif were rarely seen. We also looked at potential alterations in PLK4 and largely found a lack of alterations. Mutations in the phosphodegron of PLK4 are known to cause CA in vitro; S285 and T289 are the most important residues for βTrCP binding^11^. However, we rarely see mutations in these sites (Supplemental Fig. 3). Lastly, we looked at previously reported genes known to regulate PLK4 levels and activity. PP1 has been shown to dephosphorylate PLK4^17^. PLK4 autophosphorylates in *trans*, triggering ubiquitination by the SCF (SKP1-CUL1-F box protein) E3 ubiquitin ligase. The F-box proteins in human cells is βTrCP (BTRC). We examined these three genes, but again find a relative dearth of alterations (Supplemental Fig. 3). Therefore, PLK4 activity is likely required for CA, but genomic alterations in the core centriole duplication machinery, including PLK4, are not likely the primary drivers of CA in human cancer.

### Loss of MCPH1 is a common and penetrant cause of CA

**Figure 2:**
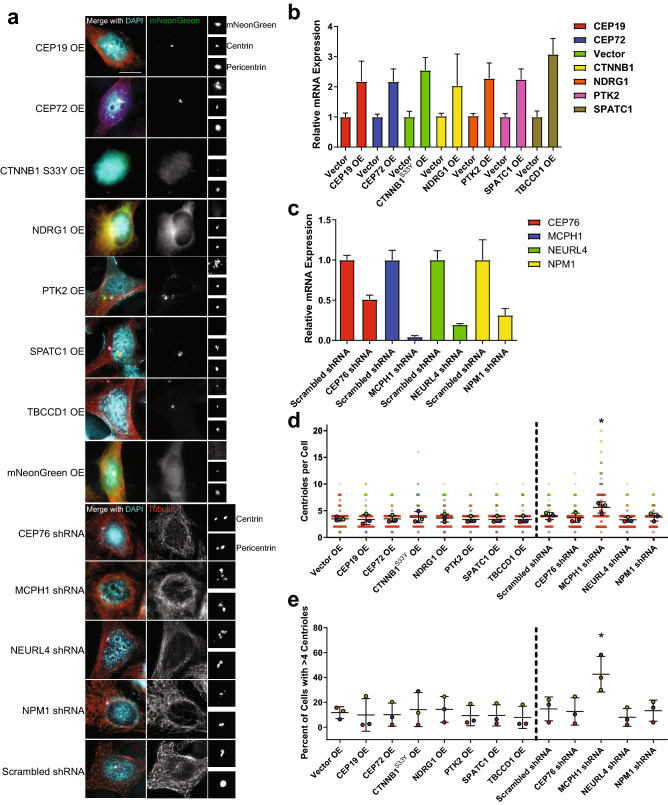
In vitro screening of lead candidates of centrosome amplification. (**a**) Representatives images of HeLa cells overexpressing mNeonGreen-tagged cDNAs corresponding to genes for which gain of function is hypothesized to cause CA, as well as shRNA-mediated depletion of genes for which loss of function is hypothesized to cause CA. The centrosome region is enlarged and shown in the images on the right side of the panel. Blue = DAPI, red = α-tubulin, green = mNeonGreen, white = pericentrin, pink = centrin. Scale bar = 5 µm. (**b**) qRT-PCR was performed to assess the amount of overexpression of the mNeonGreen-tagged genes. (**c**) qRT-PCR was performed to assess gene depletion by shRNA. For both (**b**) and (**c**), bars represent means normalized to control (vector or scrambled shRNA) ± SEM for three independent experiments. (**d**) Quantification of centrioles (centrin foci) in the indicated conditions. For cells in which a transgene was overexpressed, only cells with visible mNeonGreen expression were counted. Each larger outlined circle represents the average of technical replicates for one independent experiment, and each individual cell is shown as a more transparent dot of the same color. (**e**) Quantification of the percentage of cells with CA, defined as greater than 4 centrioles (centrin foci). Each dot represents an independent experiment. For (**d**) and (**e**), bars represent means ± SEM for three independent experiments, which include approximately 250 total cells per condition. The vertical dotted lines separate overexpression from depletion/shRNA experiments. *P value < 0.05. HeLa cells were used throughout this figure.

**Figure 3:**
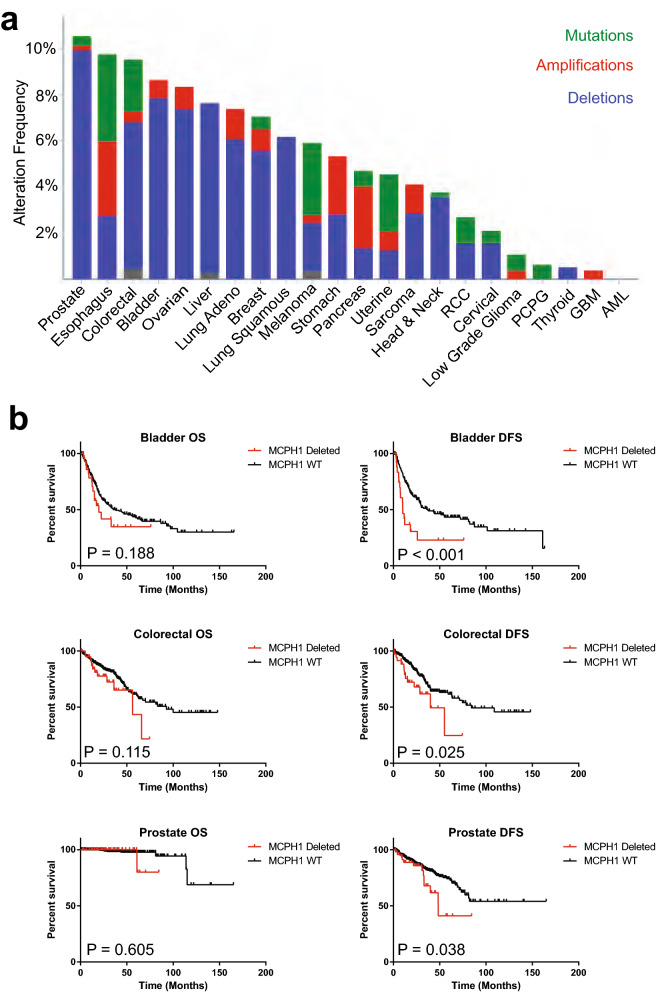
MCPH1 deletion is common in human cancer and correlates with worse outcomes. (**a**) Graphical demonstration of the percent of tumors within each tumor site that harbor deletions (blue), amplifications (red), or mutations (green). (**b**) Kaplan–Meier curves demonstrating overall survival (OS) or disease-free survival (DFS) in bladder, colorectal, and prostate cancers (some of the tumor sites where MCPH1 deletion is most common and where patient number is sufficiently high to assess survival). Displayed P values are from log-rank tests.

To test our candidate genes in vitro, we cloned and overexpressed mNeonGreen-tagged constructs of CEP19, CEP72, CTNNB1, PTK2, NDRG1, SPATC1, and TBCCD1, and utilized shRNA to deplete CEP76, MCPH1, NEURL4, and NPM1 (Fig. 2a). Because CTNNB1 point mutations are common in one particular region of the gene that is phosphorylated by GSK-3β, we also overexpressed the CTNNB1 S33Y mutant; overexpression of wild-type CTNNB1 was used as a control (Supplemental Fig. 4). Following assessment of transfection and depletion efficiency by qRT-PCR (Fig. 2b,c), we quantified centrioles in these cells. We found that loss of MCPH1 resulted in the most robust increase in centriole number (Fig. 2d,e). MCPH1 mutations are known to give rise to microcephaly and CA^18^. MCPH1 gene deletions were seen in 5–15% of human cancers, depending on the anatomic site of the tumor (Fig. 3a). Furthermore, MCPH1 deletion significantly co-occurred with p53 alterations (Bonferroni-adjusted p value < 0.001), PIDD1 alterations (p value < 0.001), and CASP2 alterations (p value = 0.007). We also assessed outcomes in patients with homozygous MCPH1 deletion. In general, there is a trend toward worse outcomes in patients with MCPH1 deletions (Table 2), and this is especially seen in bladder, colorectal, and prostate cancers (Fig. 3b). We conclude that loss of MCPH1 is common in human cancer, and depletion of MCPH1 in vitro causes CA.

**Table 2:** Survival based on homozygous MCPH1 deletion status.

Cancer site	Number of patients with homozygous deletion	Number of patients without homozygous deletion	Overall survival			Disease free survival		
			Log rank	95% CI	P value	Log rank	95% CI	P value
Bladder	26	297	1.42	0.8179–2.797	0.19	2.44	1.810–8.243	< 0.01
Prostate	49	449	1.71	0.1502–26.49	0.61	1.84	1.046–4.636	0.04
Esophagus	5	179	0.84	0.1387–5.258	0.87	17.01	113,231–1.241e8	< 0.01
Colorectal	39	507	1.6	0.8682–3.710	0.12	1.91	1.20–5.068	0.03
Ovarian	42	549	0.76	0.5330–1.149	0.21	0.93	0.6178–1.398	0.73
Lung squamous	35	466	0.95	0.5869–1.550	0.85	1.4	0.7024–3.126	0.3
Lung Adeno	30	487	0.85	0.4758–1.564	0.63	0.77	0.4364–1.440	0.45
Liver	29	341	1.66	0.9105–3.977	0.09	0.78	0.1133–4.968	0.79
Stomach	12	429	0.62	0.2740–1.689	0.41	0.23	0.1705–1.195	0.11
Breast	12	142	1.02	0.5650–1.858	0.94	0.63	0.3274–1.405	0.3
Melanoma	7	310	1.75	0.5748–7.709	0.26	1.52	0.4989–5.612	0.41
Pancreas	2	183	2.44	0.4807–36.23	0.2	1.33	0.1442–13.48	0.77
Uterine	24	515	1.61	0.7079–4.728	0.22	1.19	0.4559–3.178	0.71
Sarcoma	7	250	0.72	0.2278–2.516	0.65	0.9	0.3502–2.333	0.84
Head and neck	19	503	0.76	0.4030–1.517	0.47	1.09	0.4950–2.395	0.83
RCC	7	521	1.38	0.3848–5.559	0.58	2.11	0.5811–15.57	0.19
Cervical	7	288	3.83	1.740–138.9	0.01	3.29	0.7681–112.5	0.08
Low Grade Glioma	0	475						
PCPG	0	162						
Thyroid	2	483	0	0.001125–117.6	0.73	0	0.005156–25.94	0.64
GBM	1	571	0.81	0.1412–4.860	0.84	0.67	0.1436–3.570	0.68
AML	0	64						

### MCPH1 depletion causes centriole overduplication

**Figure 4:**
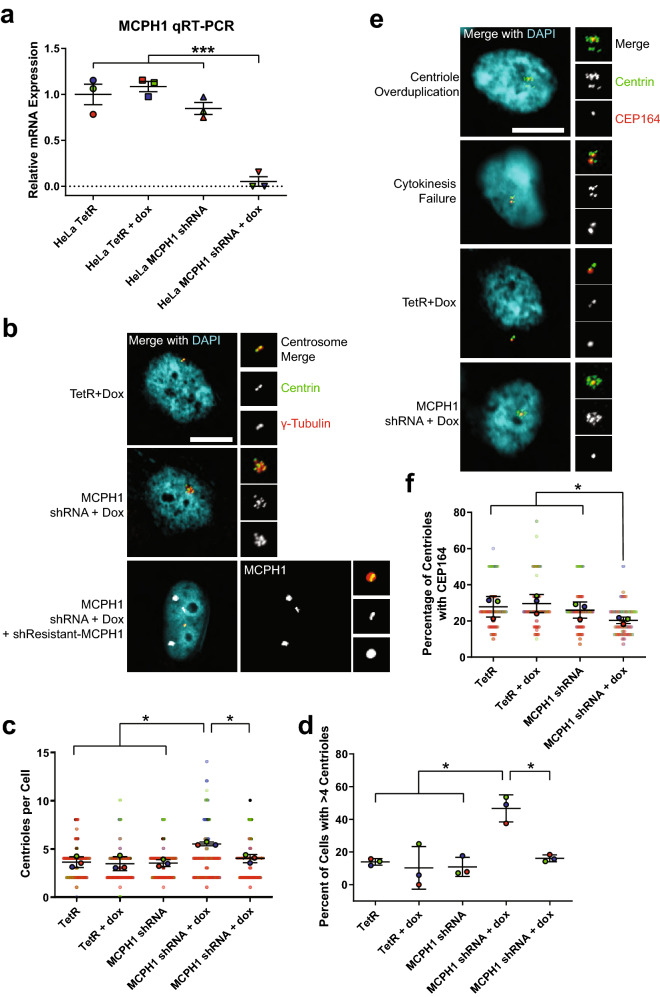
MCPH1 depletion causes centriole overduplication. (**a**) Representative images of MCPH1 qRT-PCR to assess the efficiency of doxycycline-inducible MCPH1 shRNA-mediated depletion of MCPH1 transcripts. Relative mRNA was computed by taking $2^{{ - \Delta {\text{C}}_{{\text{T}}} }}$ and normalizing to HeLa TetR. HeLa TetR is the parental cell line from which the HeLa MCPH1 shRNA cell line was made. Doxycycline was added to the indicated conditions for 24 h. Bars represent means ± SEM from 3 independent experiments, and each dot represents an independent experiment, which is color-coded to match panels (**c**) and (**e**). (**b**) Representatives image of centrioles in the indicated conditions. (**c**) Quantification of centrioles (centrin foci) in the indicated conditions. (**d**) Quantification of the percentage of cells with CA, defined as greater than 4 centrin foci. (**e**) Representative images of CEP164 staining to mark the mother centrioles. PLK4 overexpression was used as a positive control for centriole overduplication (should see lower percentage of centrioles with CEP164), and cytochalasin B induces cytokinesis failure and was used as a positive control for cell doubling (should see higher percentage of centrioles with CEP164). (**f**) Quantification of the percentage centrioles with CEP164. In panels (**c**) and (**e**), each point represents an individual cell, color-coded by independent experiment, with mean values for each experiment indicated by larger, outlined circles. Bars represent mean ± SEM from 3 independent experiments. Throughout the figure, *P value < 0.05, ***P value < 0.001, and scale bars = 5 µm.

To investigate the mechanism by which MCPH1 deletion leads to CA, we engineered a doxycycline-inducible MCPH1 shRNA HeLa cell line. Treatment with doxycycline reduced MCPH1 gene expression (Fig. 4a) and caused CA (Fig. 4b–d). This was reversed by expressing a shRNA-resistant MCPH1 transgene (Fig. 4b–d). CA can arise by two major mechanisms: (1) centriole overduplication, or (2) cell doubling (e.g. cytokinesis failure or cell–cell fusion). To determine the relative contributions of these two mechanisms, we employed a previously described technique^5^ to assess mother/mature centrioles, as the defining protein markers of mother centrioles are loaded in late G2. If centriole overduplication is the predominant mechanism, one would expect a relative dearth of mother centrioles. In contrast, if cell doubling is the predominant mechanism, one would expect to see most of the extra centrosomes containing a mother centriole. We assessed mother centrioles by staining for CEP164 and observe a smaller percentage of mother centrioles in the MCPH1-depleted cells (Fig. 4e,f). We conclude that MCPH1 deletion causes CA via centriole overduplication.

### MCPH1 depletion increases centrosomal STIL

**Figure 5:**
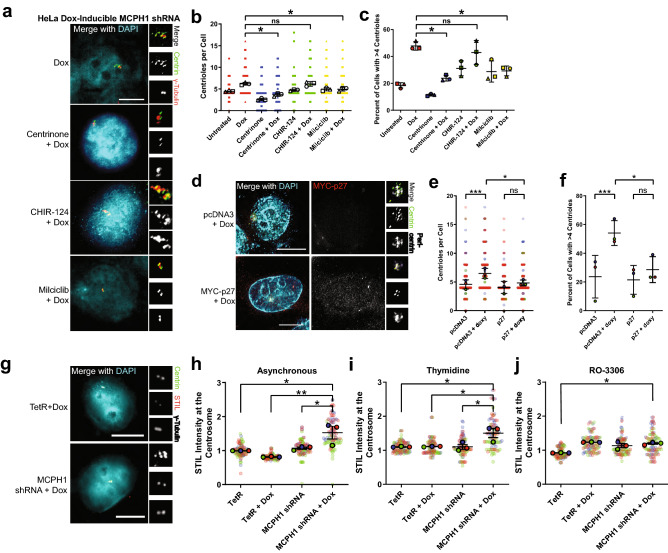
MCPH1 depletion increases STIL levels at the centrosome. (**a**) Representatives image of centrioles in the indicated conditions. (**b**) Quantification of centrioles (centrin foci) in the indicated conditions. CHIR-124 is a CHK1 inhibitor. Milciclib is a CDK2 inhibitor. (**c**) Quantification of the percentage of cells with centrosome amplification, defined as the presence of > 4 centrioles (centrin foci). Bars represent means ± SEM from 3 independent experiments. In (**b**) and (**c**), each point represents a single cell, each independent experiment is coded by shape (e.g. circle, square, triangle), and different colors are used to denote the different drug treatments (e.g. red = no additional drug, blue = centrinone, green = CHIR-124, and yellow = milciclib). The larger outlined points indicate means of each independent experiment, with bars representings mean ± SEM from the 3 independent experiments. (**d**) Cells were transfected with p27 to inhibit CDK2. (**e**,**f**) Quantification of centrioles and CA (defined as greater than 4 centrioles) in p27-transfected cells. (**g**) Representative images of STIL immunofluorescence. (**h**–**j**) Quantification of STIL immunofluorescence at the centrosome in asynchronous cells (**h**), cells arrested in S phase with thymidine (**i**), and cells arrested in G2 phase with the CDK1 inhibitor RO-3306, 10 µM (**j**). Centrosomal STIL levels were determined by measuring STIL expression within a 2.5 µm radius of the center of the centrosome and normalizing to TetR for each experiment. In (**e**), (**h**), (**i**), and (**j**), each point represents a single cell, color coded by each independent experiment, with mean values for each experiment indicated by larger, outlined circles. Bars represent mean ± SEM from 3 independent experiments. *P value < 0.05. Scale bars = 5 µm. HeLa doxycycline-inducible MCPH1 shRNA cell line is used throughout this figure.

We sought to further probe the mechanism underlying CA in the presence of MCPH1 depletion. To determine whether PLK4 is required for CA induced by MCPH1 depletion, we treated cells with centrinone B, a chemical inhibitor of PLK4^19^. Indeed, PLK4 activity is required for CA in the context of MCPH1 depletion (Fig. 5a–c).

Previous work has demonstrated that MCPH1 depletion causes loss of pericentrin from the centrosome^20^. We hypothesized that this might cause premature centriole separation, licensing centrioles for duplication. However, we do not see premature centriole splitting in our MCPH1-depleted cells. Previous work has also demonstrated that MCPH1 deficiency causes sustained CHK1 phosphorylation after ionizing radiation, which dysregulates CDK2 activity^21^. Increased CDK2 levels and activating phosphorylation at T160 are found in MCPH1-depleted cells after ionizing radiation^21^. Furthermore, CDK2 is known to be required for centriole duplication^22^, and CDK2 activity protects STIL from SCF-βTrCP-mediated degradation^16^. To test the hypothesis that MCPH1 depletion requires CDK2 to drive CA, we treated doxycycline-inducible MCPH1 shRNA cells with a chemical inhibitor of CDK2 (milciclib). CDK2 inhibition reduced CA in the context of MCPH1 depletion (Fig. 5a–c). To further substantiate the requirement for CDK2, we expressed the CDK2 inhibitor p27. Similarly, we found that p27 expression prevented CA in the context of MCPH1 depletion (Fig. 5d–f). We conclude that MCPH1-depletion causes CA that is dependent on CDK2 activity. Next, we asked whether MCPH1 depletion increased CDK2 activity or abundance using kinase and quantitative immunofluorescence assays (Supplemental Fig. 5). Kinase assays using immunoprecipitated CDK2 demonstrated no difference in CDK2 activity with changes in MCPH1 expression (Supplemental Fig. 5). Additionally, there was no difference in CDK2 abundance in MCPH1-depleted cells; this was true for whole cell and centrosomal quantification (Supplemental Fig. 5).

Centrosomal CHK1 signal is required for DNA damage-induced CA^23^, and MCPH1 deletion potentiates CHK1 activity^24^. Therefore, we tested whether CA caused by MCPH1 depletion is dependent on CHK1 activity. We treated MCPH1-depleted cells with a chemical inhibitor of CHK1 (CHIR-124). However, inhibition of CHK1 did not impair CA caused by MCPH1 depletion (Fig. 5a–c). We conclude that CA induced by MCPH1 depletion is independent of CHK1 activity. This is consistent with previous findings that CHK1 is specifically involved in CA in response to DNA damage^23^.

Given that MCPH1 depletion increases CDK2 activity^21^, and CDK2 has been shown to protect STIL from SCF-βTrCP-mediated degradation and promote STIL recruitment to the centrosome^16^, we tested the hypothesis that MCPH1 depletion increases STIL levels at the centrosome. STIL immunofluorescence was assessed before and after doxycycline-inducible depletion of MCPH1 (Fig. 5g–j). By quantitative immunofluorescence, we observed that STIL levels were increased upon MCPH1 depletion in asynchronous cells (Fig. 5h). Given that STIL levels at the centrosome change during the cell cycle, we assessed STIL levels at different phases of the cell cycle. We observed that cells arrested in S phase with thymidine displayed greater levels of STIL at the centrosome (Fig. 5i). However, STIL levels were not significantly different with arrest in G2 using the CDK1 inhibitor RO-3306 (Fig. 5j). This is perhaps not surprising, as CDK1 promotes STIL removal from the centrosome, and CDK1 inhibition stabilizes STIL at the centrosome^25^. The increased STIL expression in the context of MCPH1 depletion is specifically at the centrosome, as there were no differences seen when comparing whole cell immunofluorescence or whole cell protein expression by western blotting (Supplemental Fig. 6). These data support the notion that MCPH1 depletion causes CA, which is dependent on CDK2 activity and is associated with greater levels of STIL at the centrosome.

### Centrosome amplification caused by MCPH1 depletion depends on loss of p53

**Figure 6:**
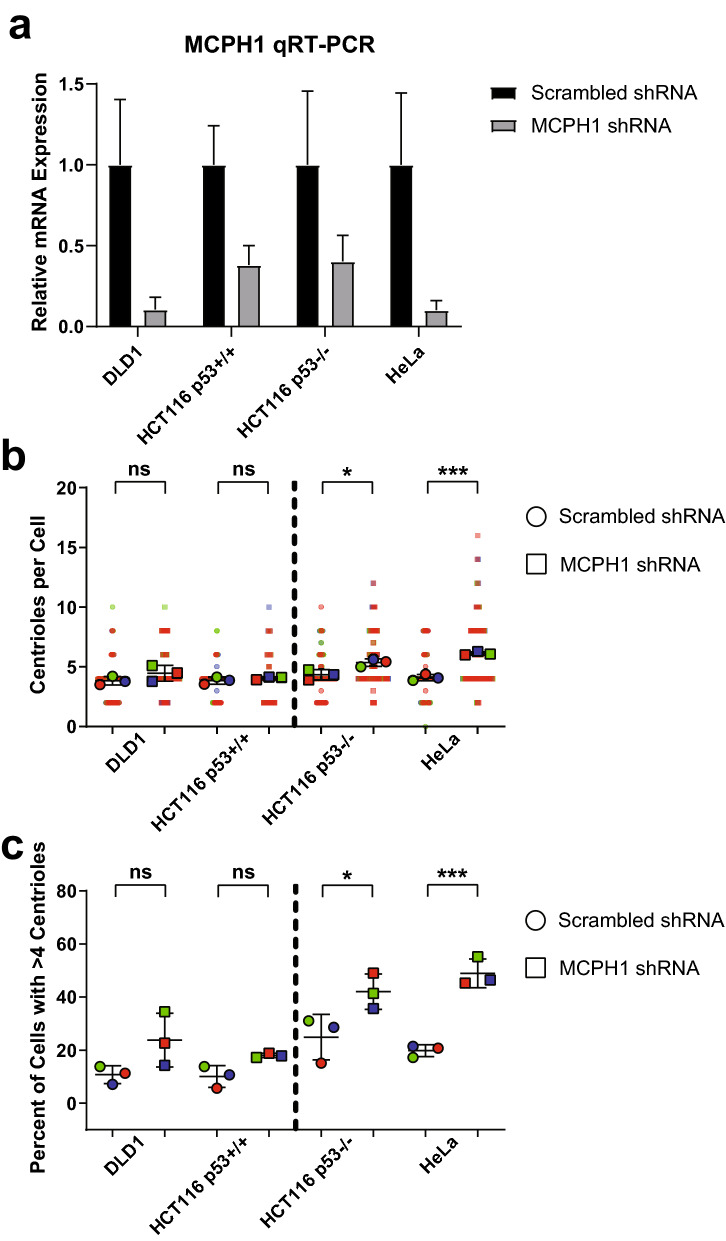
Loss of p53 permits centrosome amplification in the setting of MCPH1 depletion. (**a**) MCPH1 shRNA was used to deplete MCPH1 in, DLD1, HCT116 p53^+/+^, and HCT116 p53^−/−^, and HeLa cells. Scrambled shRNA was used as a control. (**b**) Quantification of centrioles per cell in the cells treated with shRNA. Each dot represents a single cell, color coded by each independent experiment, with mean values for each experiment indicated by larger, outlined dots. Bars represent mean ± SEM from 3 independent experiments. (**c**) Quantification of CA, defined as the percent of cells with greater than 4 centrin foci. Each dot represents a single experiment, bars represent means ± SEM. Vertical dotted lines are shown to separate cells based on p53 status; DLD1 and HCT p53^+/+^ have wild-type p53, and HCT116 p53^−/−^ and HeLa lack functional p53. *P value < 0.05, ***P value < 0.001.

Centrosome-amplified cells require additional adaptive alterations to proliferate, such as loss/mutation of p53^11^, p21^12,13^, or the PIDDosome genes (CRADD, PIDD1, CASP2)^14^. Therefore, we asked whether loss of p53 was permissive of CA in the setting of MCPH1 depletion. We used the following cell lines: HeLa, which lack functional p53 due to infection with HPV and expression of the E6 oncogene; DLD1 and HCT116 which have wild-type 53, and HCT116 p53^−/−^, in which p53 has been deleted. MCPH1 was depleted in these cell lines (Fig. 6a), which resulted in an increase in CA in all cell lines, though the increase was only statistically significant in the cell lines lacking functional p53 (HeLa and HCT116 p53^−/−^) but not in the lines with functional p53 (DLD1 and HCT116; Fig. 6b,c). We conclude that loss of p53 may be permissive of proliferation with CA in the context of MCPH1 depletion.

### Restoring MCPH1 expression reduces centrosome amplification in cancer cell lines

**Figure 7:**
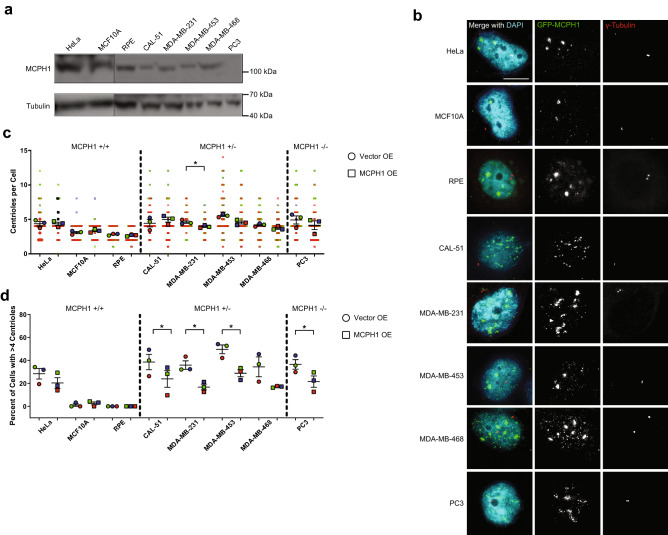
MCPH1 overexpression reduces centrosome amplification in MCPH1-deficient cancer cell lines. (**a**) Western blots demonstrating MCPH1 levels in cell lines without MCPH1 deletion (HeLa, MCF10A, and RPE), heterozygous MCPH1 deletion (CAL-51, MDA-MB-231, MDA-MB-453, and MDA-MB-468), and homozygous MCPH1 deletion (PC3). Indications are made for molecular weight markers. (**b**) Immunofluorescence images of the cell lines from panel (**a**) transfected with GFP-MCPH1 or GFP control. (**c**) Quantification of centrioles in the conditions shown in panel (**b**). Each dot represents a single cell, color coded by each independent experiment, with mean values for each experiment indicated by larger, outlined dots. Bars represent mean ± SEM from 3 independent experiments. (**d**) Quantification of CA, defined as the percentage of cells with greater than 4 centrin foci. Each dot represents a single experiment, and bars represent means ± SEM. Vertical dotted lines are shown to separate cell lines based on MCPH1 status.

A number of cancer cell lines harbor deletions in MCPH1. We next asked whether restoring MCPH1 expression in one of these cell lines could reverse CA. We used a panel of cell lines, including cell lines containing heterozygous and homozygous deletions of MCPH1 (Fig. 7a) and overexpressed MCPH1 (Fig. 7b). Overexpression of MCPH1 in the three cell lines without MCPH1 alterations, HeLa, MCF10A, and RPE, did not significantly reduce centriole number (Fig. 7c,d). In cell lines with both heterozygous and homozygous deletions of MCPH1, MCPH1 overexpression reduced CA (Fig. 7c,d). Therefore, deletion of MCPH1, either one or both copies, is responsible for CA in at least some cancer cell lines.

### Loss of MCPH1 causes chromosomal instability

Previous reports have demonstrated that loss of MCPH1 causes premature chromosome condensation, increased mitotic index, delayed chromosome alignment, multipolar spindles, and aneuploidy^20,26–31^. Accordingly, we examined the effects of MCPH1 depletion on mitosis. We found that depletion of MCPH1 increased the incidence of multipolar spindles in metaphase and lagging chromosomes and/or chromosome bridges in anaphase (Supplemental Fig. 7A–C). These data support the idea that MCPH1 deletion causes chromosomal instability, consistent with the known link between chromosomal instability and CA.

## Discussion

CA is common in human cancer, but the in vivo causes of CA have been unclear. Herein, we investigated alterations in all 367 known centrosome genes across 10,207 human cancers. In general, we identified surprisingly few genomic alterations in the centrosome genes, particularly the genes required for centriole duplication, but changes in the expression of centrosome proteins were more common, as previously reported^32,33^. This is consistent across most genes involved in mitosis^34^, in which a lack of mutations suggest that normal function of these genes are fundamental to the survival and proliferation of cancer cells. The relatively few identifiable genetic causes of CA is striking, given the high frequency of CA in cancer, suggesting that at least some causes are likely non-genetic or are indirectly caused by genetic aberrations in non-centrosome genes, which have indirect effects on CA.

However, we did identify MCPH1 deletion as one genetic cause of CA, with deep deletions present in up to 10% of cancers depending on disease site, and validated that this deletion is sufficient to cause CA in model systems. MCPH1 was first identified as a transcriptional repressor of human telomerase reverse transcriptase (hTERT)^35^ and a determinant of human brain size, with inherited mutations in MCPH1 shown to give rise to microcephaly^18,36^. MCPH1 is a *bona fide* tumor suppressor^31,37^, and many previous studies have demonstrated reduced expression in diverse cancer types, including breast, cervical, ovarian, and oral squamous cell carcinoma^29,31,38–42^. This is corroborated by murine data, in which Mcph1 knockout mice are more prone to tumorigenesis, namely lymphomas and granulosa ovary tumors^43^. This appears to be an early event, and perhaps even an initiating event, as reduced MCPH1 expression is seen in pre-malignant tissues^31^. Further, reduced expression or depletion of MCPH1 has been linked to CA, mitotic errors, metastasis, higher tumor grade and stage, and worse patient outcomes^29,31,41^. Concordant with our finding, numerous other studies have demonstrated that depletion of MCPH1 causes CA in U2OS cells^29^, MEFs^43^, and lymphoblastoid cell lines derived from human subjects with MCPH1 mutations^18^. Further, MCPH1 protein level is reduced in primary human breast cancer and is associated with CA^29^. However, one study demonstrated that Mcph1^−/−^ DT40 chicken cells have normal centrosome structure and number, though these cells exhibited much greater CA compared to their wild-type counterparts after exposure to ionizing radiation^21^. We speculate this discrepancy in DT40 cells may be due to: (1) the use of cell lines from different species; (2) the quantification of γ-tubulin foci instead of centrin foci (centrioles), a method that can be insensitive to centriole amplification; or (3) the use of acute (e.g. RNAi) versus chronic (e.g. knockout) depletion of MCPH1, as chronic depletion of MCPH1 may allow time for compensation by other centriole duplication machinery. MCPH1 deletion as a cause of CA will need to be validated in additional cohorts of primary human cancers.

MCPH1 has also been implicated in regulating the cell cycle. Mcph1 knockout murine neural progenitor cells demonstrate uncoupling of the centrosome cycle from the cell cycle^45^. Our study also revealed MCPH1 deletion as a cause of chromosomal instability in human cancer, as demonstrated by frequent aberrant mitoses. This is consistent with the following previous reports: (1) Mcph1 knockout mouse neuroprogenitor cells exhibit more mitotic spindle defects, including multipolar spindles^45^; (2) Mcph1 knockout mouse embryonic fibroblasts (MEFs) exhibit more metaphase aberrations in response to ionizing radiation compared to wild-type MEFs^46^; (3) U2OS cells treated with MCPH1 siRNA also exhibit more mitotic aberrations, namely chromosome missegregation, disorganized spindles, and cytokinesis failure^29^; and (4) HeLa cells treated with MCPH1 siRNA exhibit premature chromatin condensation prior to mitosis and longer time from nuclear envelope breakdown to chromosome alignment in metaphase. In addition to chromosomal instability, MCPH1 is critical for protecting genome stability, as it helps mediate intra-S and G2 arrest after DNA damage^30,47^. Mechanistically, MCPH1 is important for recruiting RAD51/BRCA2 to sites of DNA damage^46^. MCPH1 may also be important for gametogenesis, as Mcph1-knockout mice are infertile and exhibit meiotic defects^46^.

MCPH1 is on chromosome 8p23.1, a region of the genome that is commonly deleted in human cancer, is occasionally associated with gains of chromosome 8q, and portends worse outcomes^49^. Most of the deletions we observed in MCPH1 are coincident with larger 8p deletions. This begs the question: deletion of which gene or genes on 8p confers the tumorigenicity of 8p deletion? Herein we provide evidence that one mediator of the phenotype observed in 8p-deleted cancers is MCPH1, which may cause CA and chromosomal instability in 8p-deleted cancers. However, one caveat to our study is that MCPH1 deletion may be a passenger event rather than a driving event in human cancer. Another question raised is whether loss of one copy of MCPH1 is sufficient to cause CA. Previous work has shown that Mcph1^+/−^ MEFs exhibit CA, and Mcph1^−/−^ MEFs have even greater CA^43^. Furthermore, our data showing that overexpression of MCPH1 reduces CA in cell lines with heterozygous MCPH1 deletion suggest that deletion of one copy of MCPH1 is indeed sufficient to cause CA, albeit to a lesser extent than homozygous MCPH1 deletion.

Proliferation in the presence of CA requires additional adaptations, such as loss/mutation of p53^11^, p21^12,13^, or the PIDDosome genes^14^. In tumors with MCPH1 deletion, we observed that approximately 55% also had one of the above compensatory alterations compared to approximately 40% of tumors without MCPH1 deletion. Therefore, there are likely other potential compensatory alterations that are permissive of cellular proliferation in the context of CA. Previous work has also shown that p53 knockout is more permissive of CA with Mcph1 depletion in MEFs^43^. Mechanistically, MCPH1 may contribute to p53 stability by inhibiting MDM2-mediated ubiquitination and degradation of p53^37^. In our study, we found that depletion of MCPH1 results in CA in cell lines lacking functional p53, but the effect was not as profound in cell lines with functional p53. However, one caveat to this is that DLD1, which lacks a p53 mutation, silences wild-type p53 and is capable of proliferating with CA or loss of centrioles^50,51^. Therefore, MCPH1 depletion may not uniformly cause CA in all cell lines or contexts.

Our study focused mostly on causes of numerical CA. However, structural centrosome abnormalities have also been reported, such as increased centriole length. Increased centriole length is caused by CPAP overexpression or CP110 depletion^52^, but according to our analyses, neither genomic amplification nor mRNA overexpression/underexpression are commonly seen in human cancer. Increased centrosome size can be caused by overexpression of NLP/NINL^53^ and other mediators of microtubule nucleation, but our analyses demonstrate that neither genomic amplification nor mRNA overexpression are common. Increased centrosome size can also be caused by increased centriole number or length^6^, and the relative contribution of all these mechanisms to centrosome size remain unclear. Another potential limitation is that our study only focused on genes with reported localization to the centrosome; however, we know that other extra-centrosomal genes, such as transcriptional regulators or post-translational modifiers, can alter the levels of centrosome proteins and could drive CA. Lastly, inhibition of cell cycle progression can lead to CA, and this would not have been detected by our study.

Despite our in-depth analysis of centrosome gene alterations, there still remains a significant fraction of CA that is unaccounted for by genomic alterations. There are a number of possible explanations. First, there could be mutations affecting gene regulatory regions that influence chromatin structure or gene expression levels that might not have been discovered by exome sequencing alone. Secondly, there could be changes in non-protein coding RNAs that regulate the levels of centrosome genes. Thirdly, there could be post-transcriptional and/or post-translational regulation of mediators of centriole duplication that were undetermined by our screen. Finally, the cause of CA could be non-genetic and heritable, as supported by some previous work on centriole amplification or cell doubling^54^. Another caveat to our study is that our overexpression of candidate genes was limited; however, we addressed this by only analyzing centrosomes in cells with observable GFP expression. Another caveat is that we did not directly determine whether the increased centrosomal STIL in MCPH1-depleted cells is a consequence or a cause of CA.

## Conclusions

The goal of this project was to determine the causes of CA in human cancer. Our analysis of alterations in centrosome genes and in vitro validation of the most common alterations demonstrated that MCPH1 deletion occurs in up to 10% of human cancers, depending on the disease site, and causes CA. Furthermore, the mechanism of MCPH1 depletion-mediated CA depends on increased CDK2 activity and is associated with increased levels of STIL at the centrosome. This study provides additional insight into the clinically-relevant causes of CA in human cancer, revealing MCPH1 deletion as a common and penetrant driver of CA. It is conceivable that there are other drivers of CA that warrant future investigation.

## Supplementary information

Supplementary Information 1.

Supplementary Information 2.

## Data Availability

The datasets generated during and/or analyzed during the current study are available from the corresponding author on reasonable request.
