# Supplementary material for: Analysis of the “centrosome-ome” identifies MCPH1 deletion as a cause of centrosome amplification in human cancer
Source: Sci Rep. 2020 Jul 17;10:11921. doi: 10.1038/s41598-020-68629-4 (PMC7368085; doi:10.1038/s41598-020-68629-4)

SUPPLEMENTAL DATA

Analysis of the "centrosome-ome" identifies MCPH1 deletion as a cause of centrosome amplification in human cancer

[1,2,3]Ryan A. Denu, [1,2]Mark E. Burkard

Author Affiliations:

1. Division of Hematology/Oncology, Department of Medicine, School of Medicine and Public Health, University of Wisconsin-Madison, Madison, WI, USA
2. Carbone Cancer Center, University of Wisconsin, Madison, WI, USA
3. Medical Scientist Training Program, School of Medicine and Public Health, University of Wisconsin-Madison, Madison, WI, USA

Corresponding Author: Mark Burkard, University of Wisconsin-Madison, 6059 WIMR, 1111 Highland Avenue, Madison, WI 53705, Phone: (608) 262-2803, email: meburkard@medicine.wisc.edu

Supplemental Table 1. List of centrosome genes included in analysis.

Supplemental Table 2. Enrichment of centrosome gene alterations in p53 mutant/deleted tumors.

Supplemental Table 3. Analysis of genes required for centriole duplication.

Supplemental Table 4. Primer sequences for qRT-PCR.

Supplemental Figure 1. NPM1 mutations in acute myeloid leukemia (AML) do not cause centrosome amplification.

(A) Diagram of NPM1 mutations in TCGA datasets using cBioPortal to extract and display the data. The most common alteration is a frameshift at the C terminus. **(B)** Kaplan-Meier curve demonstrating overall survival in AML patients with mutant NPM1 versus wild type NPM1. **(C)** Kaplan-Meier curve demonstrating disease-free survival in AML patients with mutant NPM1 versus wild type NPM1. P values from log rank tests are displayed. **(D)** Oncoprint of NPM1 alterations along with genes whose loss is permissive of proliferation in the setting of centrosome amplification (TP53, CDKN1A, PIDD1, CRADD, and CASP2). There is a lack of coincidence of NPM1 mutation with loss of these other genes. **(E)** Representative images of HeLa cells transfected with either wild type or mutant NPM1. **(F)** Quantification of centrioles (centrin foci) per cell in the indicated conditions. Each dot represents one cell and bars represent means ± SD. **(G)** Quantification of the percentage of cells with centrosome amplification, defined as greater than 4 centrioles (centrin foci). Bars represent means ± SEM. NS = not significant.

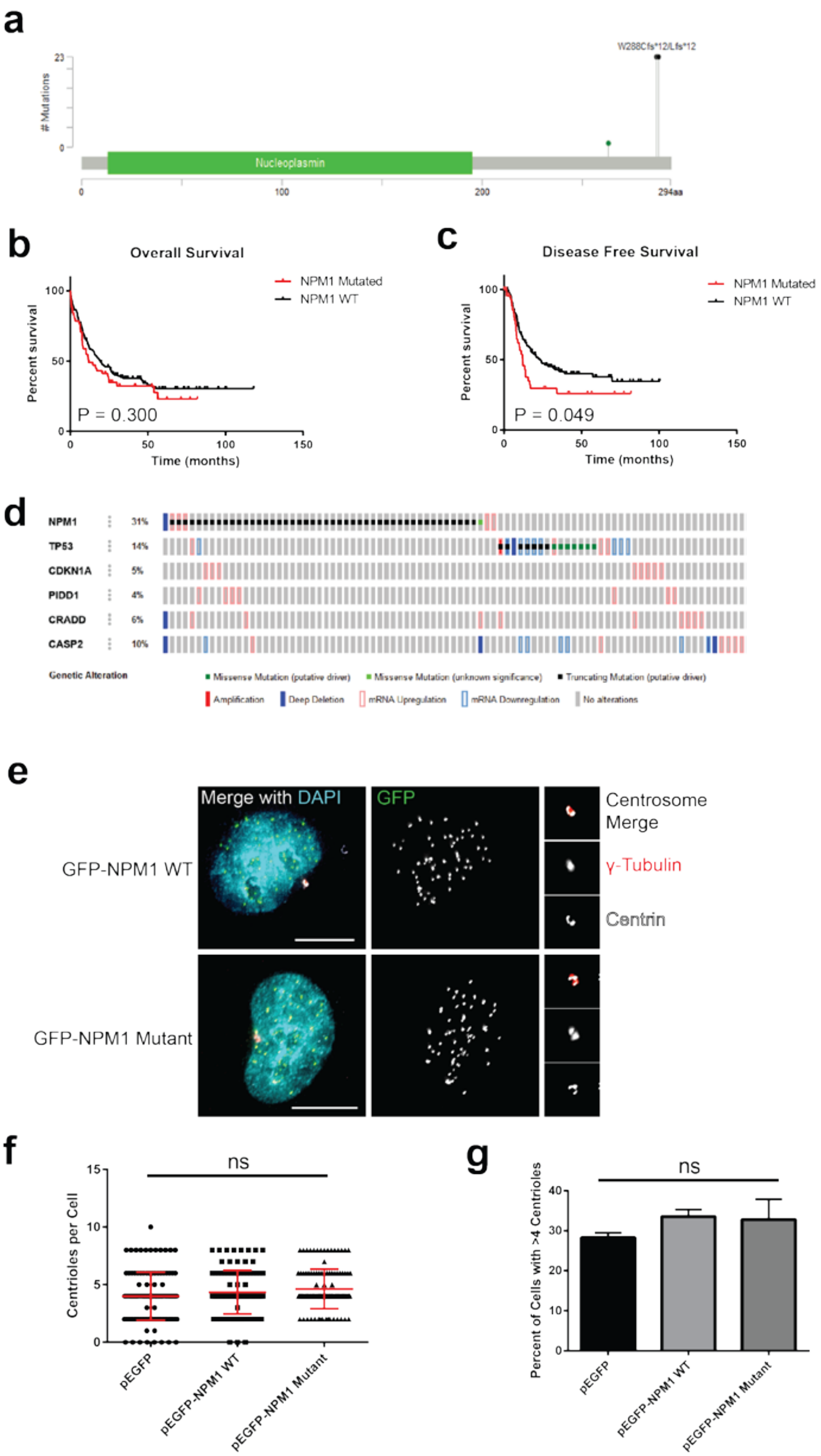
a
W288Cfs*12/Lfs*12
Mutations
23
0
Nucleoplasmin
0
100
200
294aa
b
Overall Survival
NPM1 Mutated
NPM1 WT
Percent survival
P = 0.300
Time (months)
c
Disease Free Survival
NPM1 Mutated
NPM1 WT
Percent survival
P = 0.049
Time (months)
d
NPM1 31%
TP53 14%
CDKN1A 5%
PIDD1 4%
CRADD 6%
CASP2 10%
Genetic Alteration
Missense Mutation (putative driver)
Missense Mutation (unknown significance)
Truncating Mutation (putative driver)
Amplification
Deep Deletion
mRNA Upregulation
mRNA Downregulation
No alterations
e
Merge with DAPI
GFP
Centrosome Merge
γ-Tubulin
Centrin
GFP-NPM1 WT
GFP-NPM1 Mutant
f
ns
Centrioles per Cell
pEGFP
pEGFP-NPM1 WT
pEGFP-NPM1 Mutant
g
ns
Percent of Cells with >4 Centrioles
pEGFP
pEGFP-NPM1 WT
pEGFP-NPM1 Mutant

Supplemental Figure 2. Investigation of alterations in CPAP and CP110.

(A) Mutation diagram and prevalence of genomic alterations in CPAP. **(B)** Mutation diagram and prevalence of genomic alterations in CP110. Data were extracted using cBioPortal.

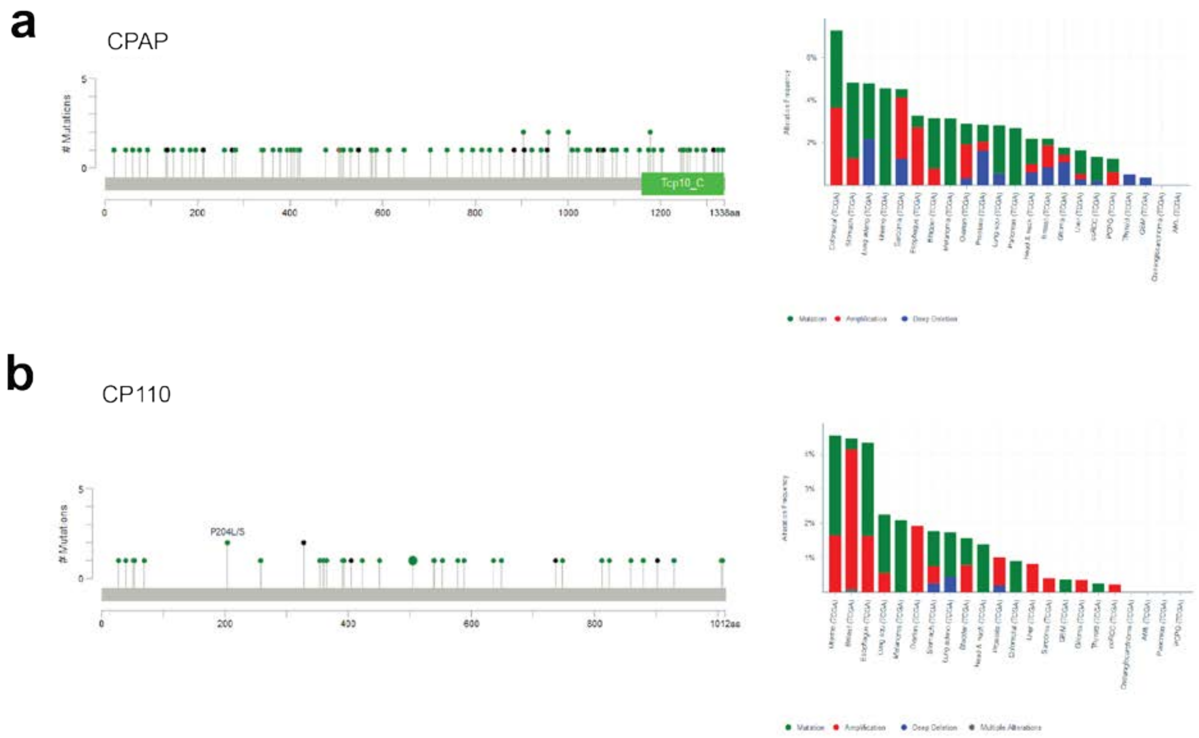

Supplemental Figure 3. Investigation of alterations in PLK4 and its regulators.

(A) Mutation diagram and prevalence of genomic alterations in PLK4, the master regulator of centriole duplication. **(B)** Mutation diagram and prevalence of genomic alterations in SKP1, which helps degrade autophosphorylated PLK4. **(C)** Mutation diagram and prevalence of genomic alterations in CUL1, which helps degrade autophosphorylated PLK4. **(D)** Mutation diagram and prevalence of genomic alterations in BTRC, which helps degrade autophosphorylated PLK4. Green = mutations; red = amplifications; blue = deletions. Data were extracted using cBioPortal.

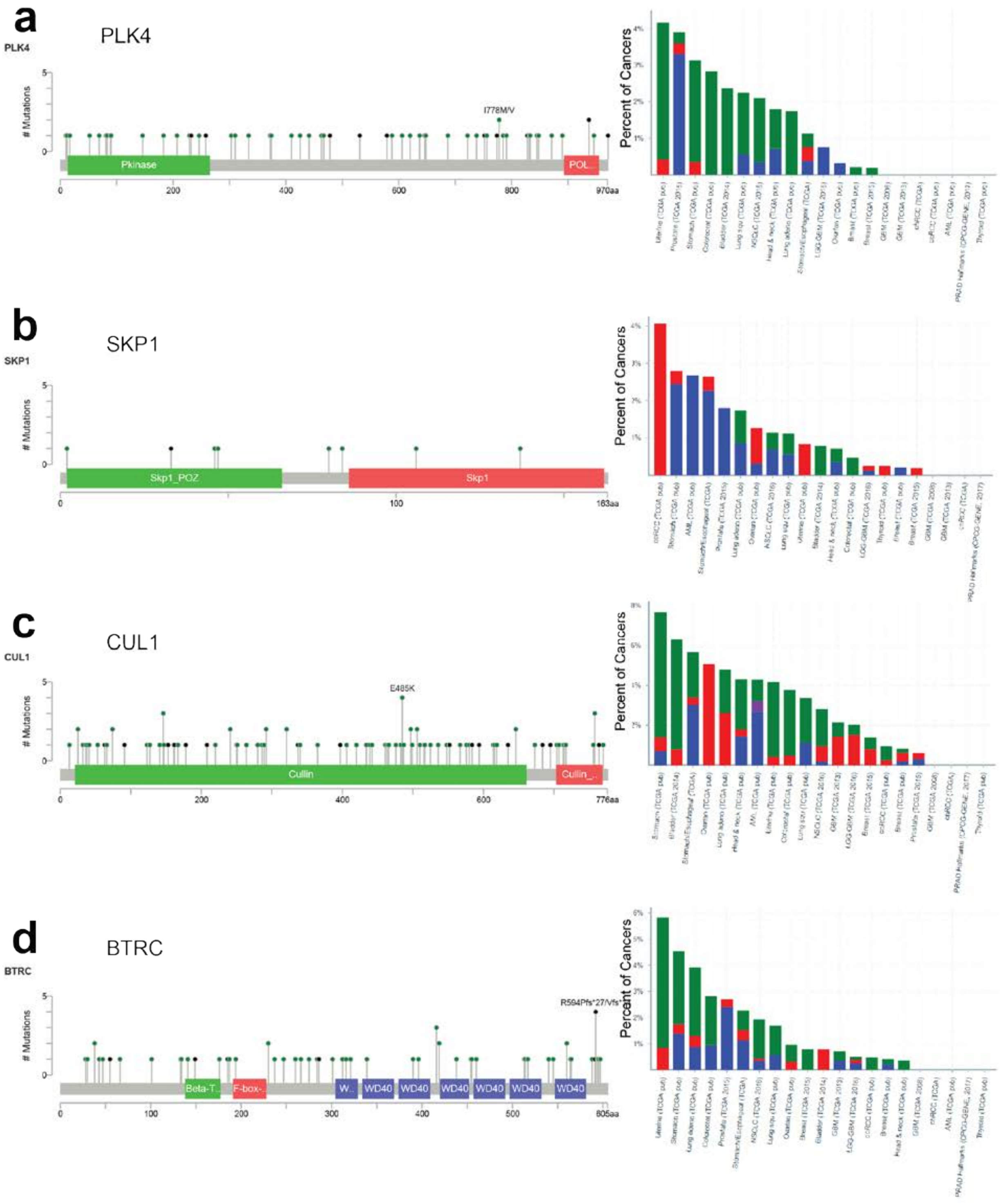
a
PLK4
Mutations
I778M/V
Pkinase
POL...
970aa
Percent of Cancers
b
SKP1
Mutations
Skp1_POZ
Skp1
163aa
Percent of Cancers
c
CUL1
Mutations
E485K
Cullin
Cullin_...
776aa
Percent of Cancers
d
BTRC
Mutations
R594Pfs*27/Vfs*
Beta-T...
F-box-...
W...
WD40
805aa
Percent of Cancers

Supplemental Figure 4. Overexpression of CTNNB1 does not cause centrosome amplification.

(A) Representative images of HeLa cells transfected with pcDNA vector, wild type CTNNB1, or CTNNB1 S33Y. Scale bars = 5µm. **(B)** Quantification of centrioles (centrin foci) in the indicated conditions. Each dot represents one cell. Bars represent means ± SD.

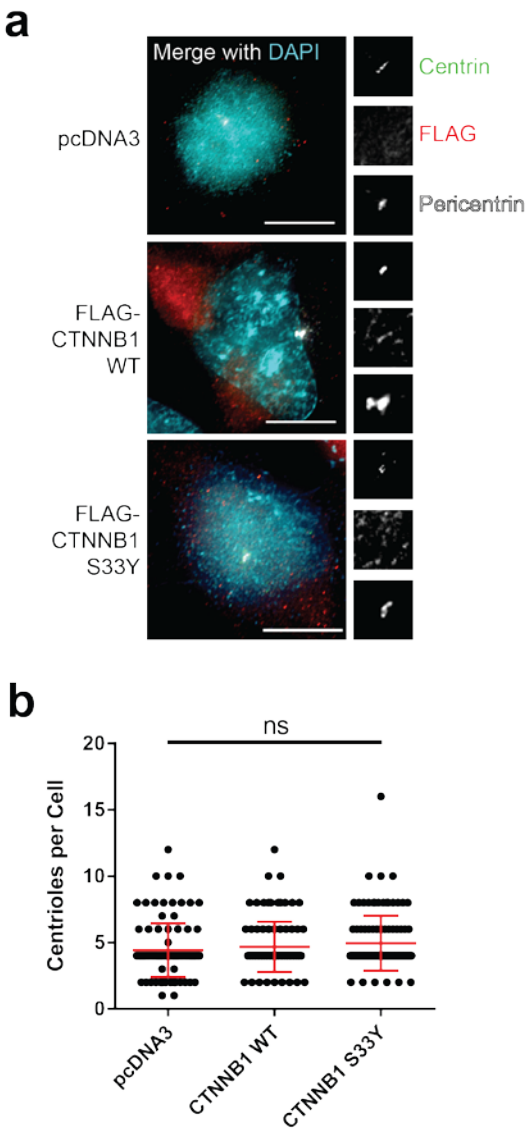

Supplemental Figure 5. CDK2 activity and expression and whole-cell STIL expression are not altered by MCPH1 depletion.

(A) Kinase assay using CDK2 immunoprecipitated from the indicated cell conditions. Anti-FLAG antibody was used as a control (“Control Ab”). Milciclib is a CDK2 inhibitor. Histone H1 was used as the substrate. **(B-C)** Quantification of CDK2 intensity at the centrosome (B) and in the entire cell (C) in the indicated conditions. **(D)** Quantification of whole cell STIL immunofluorescence in asynchronous cells. **(E)** Western blotting for STIL expression using whole-cell extracts. Indications are made for molecular weight markers. Throughout the figure, each dot represents a single cell, bars represent means ± SD, ns = not significant.

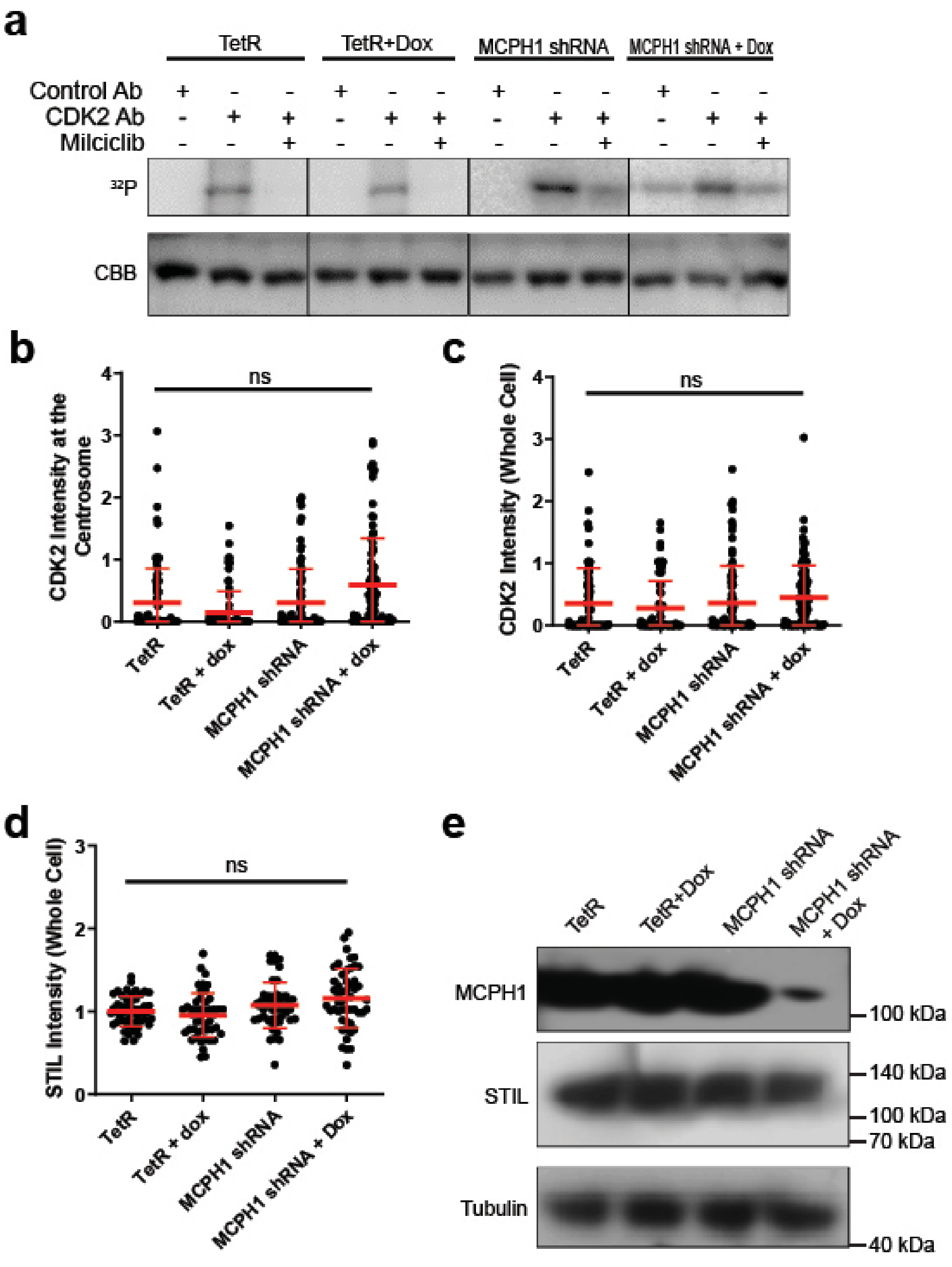
a
TetR
TetR+Dox
MCPH1 shRNA
MCPH1 shRNA + Dox
Control Ab
CDK2 Ab
Milciclib
32P
CBB
b
CDK2 Intensity at the Centrosome
ns
TetR
TetR + dox
MCPH1 shRNA
MCPH1 shRNA + dox
c
CDK2 Intensity (Whole Cell)
ns
TetR
TetR + dox
MCPH1 shRNA
MCPH1 shRNA + dox
d
STIL Intensity (Whole Cell)
ns
TetR
TetR + dox
MCPH1 shRNA
MCPH1 shRNA + Dox
e
TetR
TetR+Dox
MCPH1 shRNA
MCPH1 shRNA + Dox
MCPH1
STIL
Tubulin
100 kDa
140 kDa
100 kDa
70 kDa
40 kDa

Supplemental Figure 6. MCPH1 depletion causes genomic instability.

(A) HeLa cell lines were fixed with methanol, stained, and analyzed. The images depict representatives of the different mitotic phenotypes that were assessed. Scale bars = 5 µm. ACA = anti-centromere antibody (CREST). **(B)** Quantification of metaphase cells as either normal bipolar, multipolar, monopolar, or misaligned (includes polar chromosomes). **(C)** Quantification of anaphase cells as either normal bipolar, lagging or bridge chromosomes, or multipolar. Bars represent means ±SEM from 3 independent experiments of at least 100 metaphase cells and 50 anaphase cells in each experiment (i.e. total n > 300 metaphase cells and >150 anaphase cells). *P value < 0.05 for 2-way ANOVA interaction.

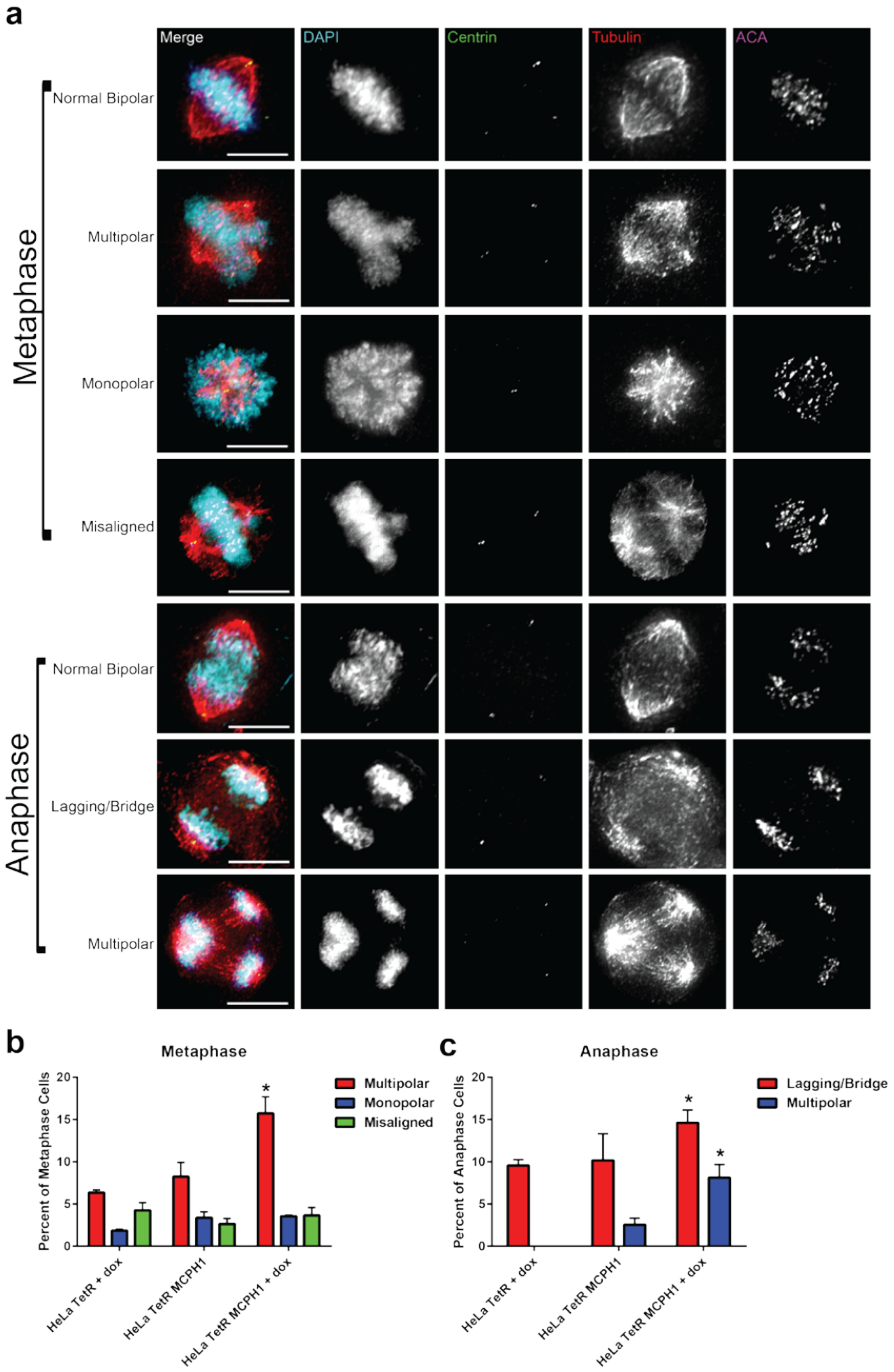
a
Merge
DAPI
Centrin
Tubulin
ACA
Metaphase
Normal Bipolar
Multipolar
Monopolar
Misaligned
Anaphase
Normal Bipolar
Lagging/Bridge
Multipolar
b
Metaphase
Percent of Metaphase Cells
Multipolar
Monopolar
Misaligned
HeLa TetR + dox
HeLa TetR MCPH1
HeLa TetR MCPH1 + dox
c
Anaphase
Percent of Anaphase Cells
Lagging/Bridge
Multipolar
HeLa TetR + dox
HeLa TetR MCPH1
HeLa TetR MCPH1 + dox

Supplemental Figure 8. Uncropped immunoblots shown in figures.

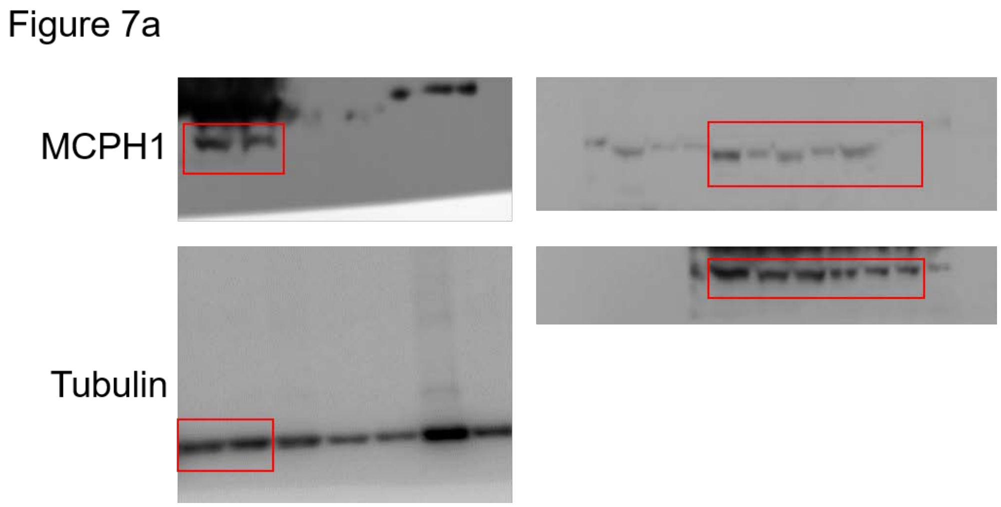

Supplement: Supplementary file 1 — Supplementary Information 1. [file 41598_2020_68629_MOESM1_ESM.pdf]
